# Supplementary material for: A broad-spectrum virus- and host-targeting peptide against respiratory viruses including influenza virus and SARS-CoV-2
Source: Nat Commun. 2020 Aug 25;11:4252. doi: 10.1038/s41467-020-17986-9 (PMC7447754; doi:10.1038/s41467-020-17986-9)
Supplement: Supplementary file 1 — Supplementary information [file 41467_2020_17986_MOESM1_ESM.pdf]

Supplementary information

A broad-spectrum virus- and host-targeting peptide against respiratory viruses including influenza virus and SARS-CoV-2

Hanjun Zhao, Kelvin K. W. To, Kong-Hung Sze, Timothy Tin-Mong Yung, Mingjie Bian, Hoiyan Lam, Man Lung Yeung, Cun Li, Hin Chu, Kwok-Yung Yuen

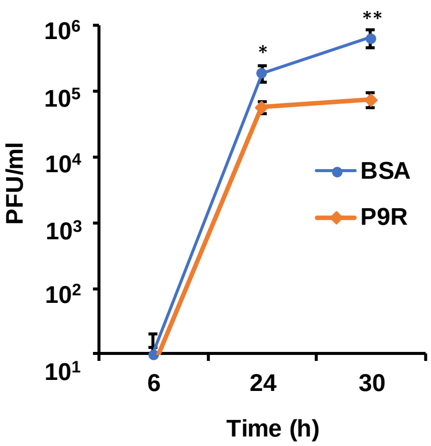

Supplementary Fig. 1. P9R could significantly inhibit SARS-CoV-2 replication at post infection time. VERO-E6 cells were infected with SARS-CoV-2 (0.005 MOI) and then P9R or BSA (50 µg ml-1) was added to VERO-E6 cells at 6 h and 24 h post infection. The supernatants were collected at 6 h, 24 h and 30 h post infection. The viral titers in the supernatants were measured by plaque assay. * indicates P=0.011 and ** indicates P=0.007when compared with BSA. P values were calculated by the two-tailed Student's t test. Data are presented as mean ±SD from three independent experiments.

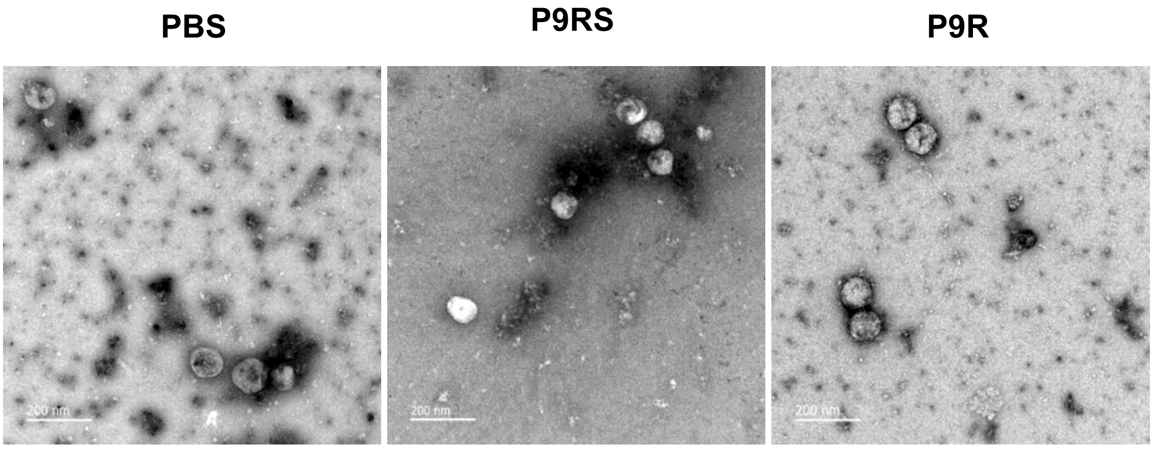

Supplementary Fig. 2. P9R did not show activity to disrupt the viral particles. P9R or P9RS (200 µg ml$_{-1}$) were premixed with SARS-CoV-2 for incubation at room temperature for 1h. Then the viral particles were fixed by formalin for negative staining. The represent negative staining pictures were taken by by FEI Tecnal G2-20 TEM from three biologically independent samples. Scar bar: 200 nm.

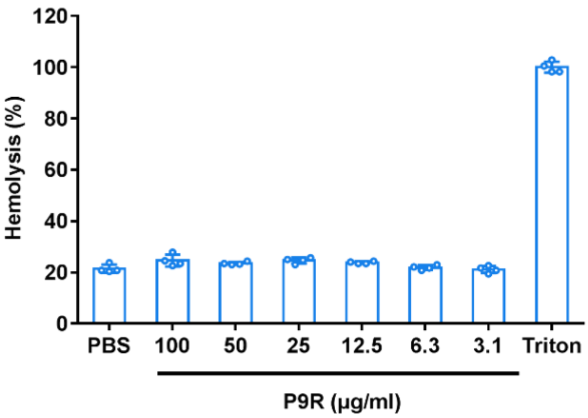

Supplementary Fig. 3. P9R did not cause the hemolysis of RBCs. Chicken red blood cells (RBCs) were pretreated by P9R at the indicated concentrations. PBS was as the negative control and Triton X-100 (0.1%) was as the positive control of RBC hemolysis. After 1h incubation at 37 °C, the treated RBCs were centrifuged at 350g for 3 min. The supernatants were measured at 450 nm for the hemolysis assay. Data are presented as mean ±SD from four biologically independent samples.

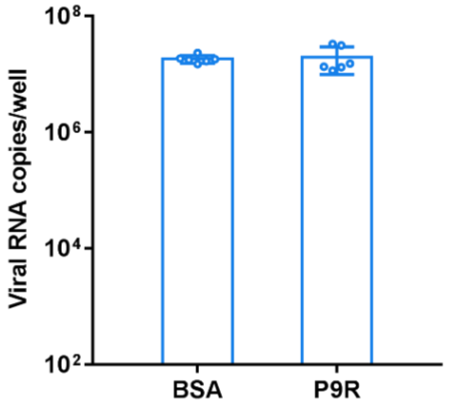

Supplementary Fig. 4. P9R did not show antiviral activity before viral infection. MDCK cells were pretreated by P9R or BSA (25 μg ml-1) at room temperature for 45 min. After washing the peptides, H1N1 virus (0.1 MOI) infected the treated cells. Viral RNA copies were measured at 5 h post infection. Data are presented as mean ±SD from three independent experiments.

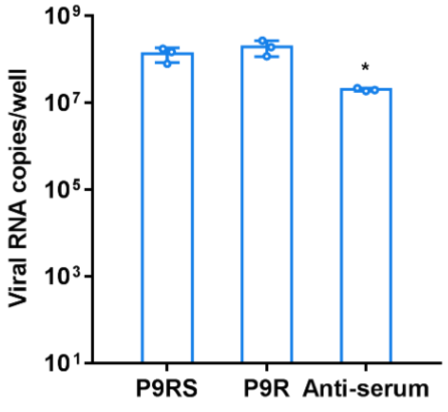

Supplementary Fig. 5. P9R did not affect the viral attachment on cells. H1N1 virus (1 MOI) were pretreated by P9RS, P9R (50 μg ml-1) or anti-serum at room temperature for 45 min. Virus and cells were cold on ice for 15 min and then the cold virus was added to cells for viral attachment on ice for 1h. The attached virus was measured by RT-qPCR. * indicates P=0.016 when compared with P9RS. P values were calculated by the two-tailed Student's t test. Data are presented as mean ±SD from three independent experiments.

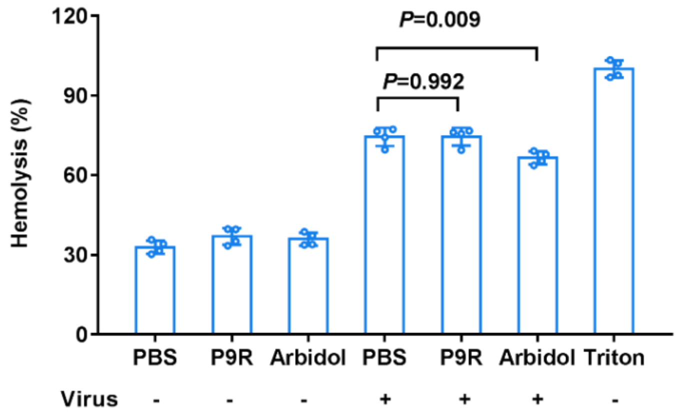

Supplementary Fig. 6. P9R did not inhibit virus-induced hemolysis. P9R (200 μg ml-1) or Arbidol (100 μg ml-1) were mixed with same volume of H1N1 virus (HA titer >128) or PBS for 1 h, and then 60 μL of 2% chicken red blood cells was added for 15 min incubation. PBS and Triton X-100 (0.1%) were included as the negative and positive control of hemolysis. The precipitated erythrocytes were incubated with sodium citrate solution (pH of 4.9) for 25 min before the hemolysis measure. *P* values were calculated by the two-tailed Student's t test. Data are presented as mean ±SD from four biologically independent samples.

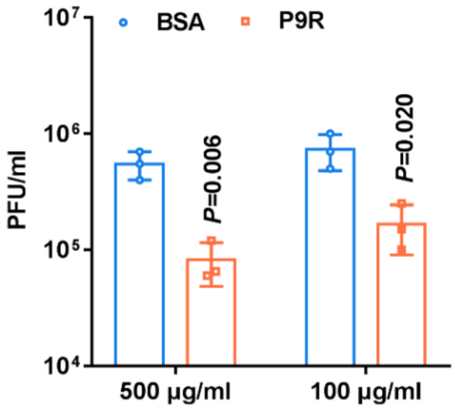

Supplementary Fig. 7. P9R showed irreversible antiviral activity after P9R binding to virus. H1N1 virus (~1×106 PFU/ml) was pretreated by P9R or BSA (500 μg/ml or 100 μg/ml) and incubated at room temperature for 45 min. The virus-peptide mixtures were serially diluted for plaque assay. After 10, 000-fold dilution, P9R (0.05 μg/ml or 0.01 μg/ml) could significantly inhibit viral replication when compared with BSA-treated virus. *P* values were calculated by the two-tailed Student's t test when compared with BSA. Data are presented as mean ±SD of three independent experiments.

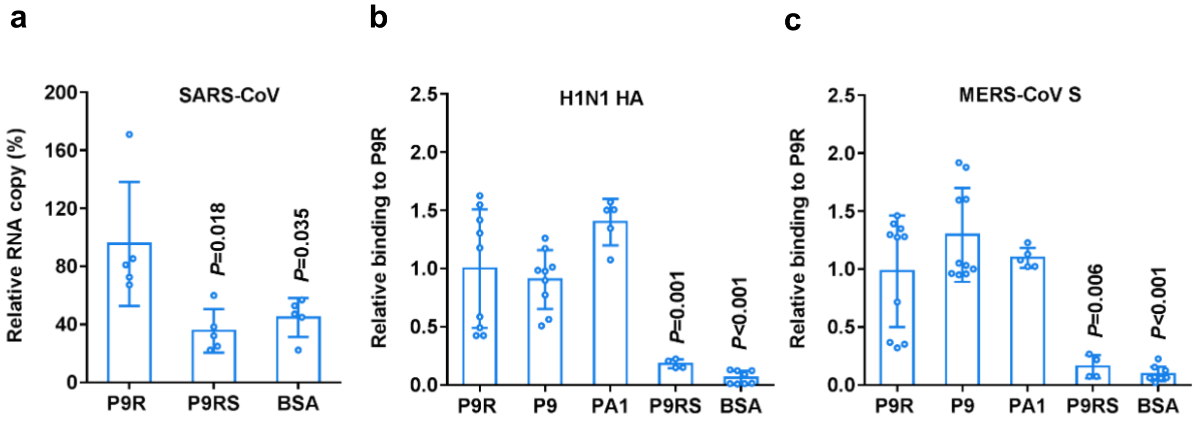

Supplementary Fig. 8. Peptides binding to virus and viral proteins. (**a**) Peptides binding to SARS-CoV (n=5). SARS-CoV was incubated with the indicated peptides on ELISA plate for 1h. The unbound virus was washed away and the bound SRAR-CoV was quantified by RT-qPCR. Relative RNA copy (%) was normalized to RNA copy of virus binding to P9R. (**b**) Peptides binding to H1N1 HA1 protein (n=9). (**b**) Peptides binding to MERS-CoV S protein (n=10). Peptides were coated on ELISA plates. The H1N1 HA and MERS-CoV S proteins binding to peptides were measured by ELISA assay. *P* values were calculated by the two-tailed Student's t test when compared with P9R. Data are presented as mean ±SD of at least three independent experiments.

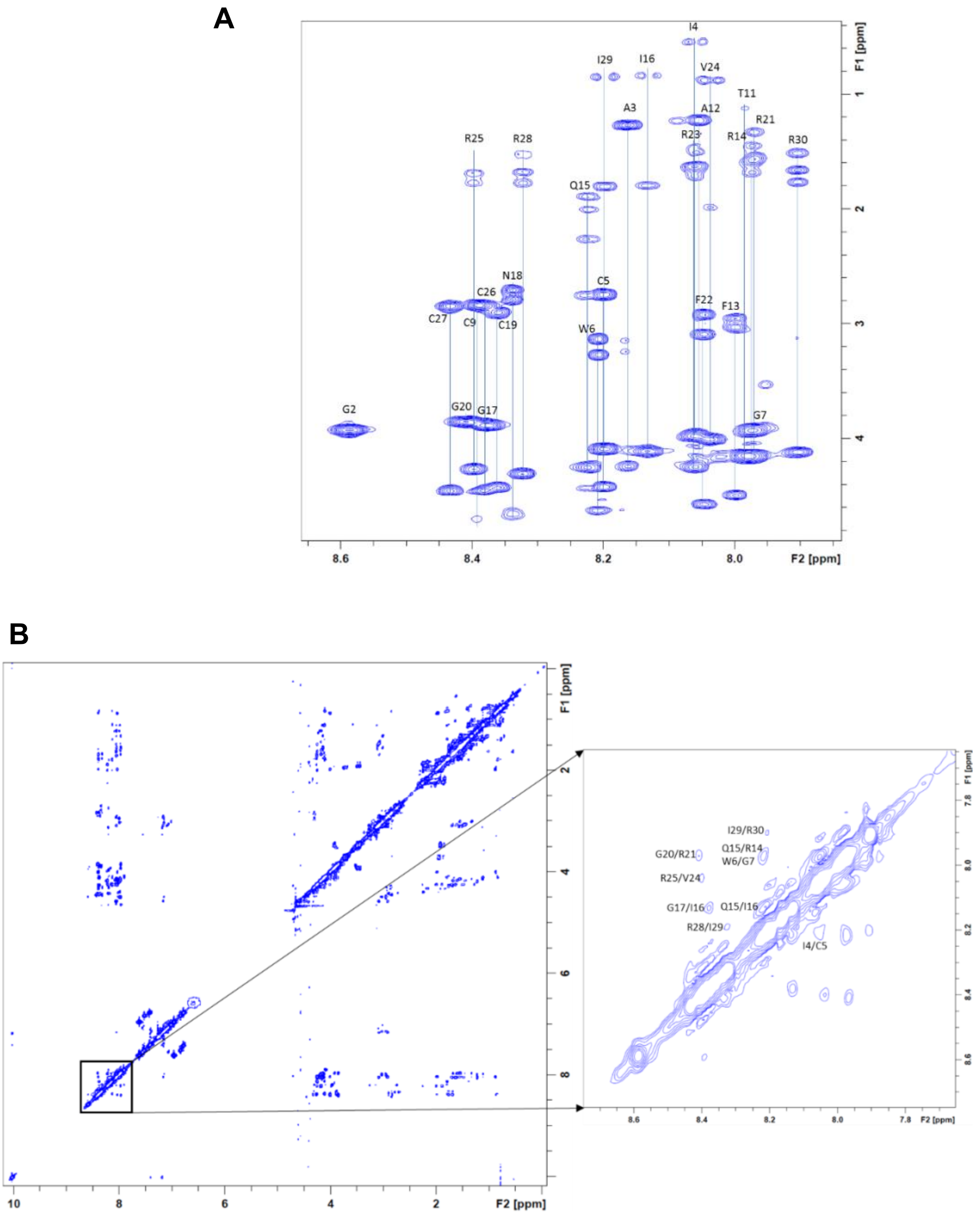

Supplementary Fig. 9. The NMR spectrum of P9R. (**a**) Fingerprint region of 2D TOCSY spectrum, showing amide cross peak region of peptide P9R. (**b**) 2D NOESY spectrum of peptide P9R at a mixing time of 300 ms. The expanded insert shows the NH-NH region.

Supplementary Table 1. NMR and refinement statistics for P9R

	**P9R (PDB 6M56)**
**NMR distance and dihedral constraints**	
Distance constraints	
Total NOE	669
Intra-residue	450
Inter-residue	137
Sequential ($\|i-j\| = 1$)	128
Medium-range ($2 \le \|i-j\| \le 4$)	9
Long-range ($\|i-j\| \ge 5$)	0
Intermolecular	0
Hydrogen bonds	0
Total dihedral angle restraints	40
ϕ	**20**
ψ	**20**
**Structure statistics**	
Violations (mean and s.d.)	
Distance constraints (Å)	0.21 ± 0.071
Dihedral angle constraints (º)	0
Max. dihedral angle violation (º)	0
Max. distance constraint violation (Å)	0.29
Deviations from idealized geometry	
Bond lengths (Å)	0.0027 ± 0.00011
Bond angles (º)	0.37 ± 0.017
Impropers (º)	0.93 ± 0.13
Average pairwise r.m.s. deviation** (Å)	
Heavy (residues 3-5, 8-16, 21-28)	5.02 ± 1.10
Backbone (residues 3-5, 8-16, 21-28)	4.18 ± 0.97

**Pairwise r.m.s. deviation was calculated among 20 refined structures.

Supplementary Table 2. RT-qPCR primers

Gene	Primer	Oligonucleotide sequence (5' to3')
SARS-CoV-2	S-F	CCTACTAAATTAAATGATCTCTGCTTTACT
	S-R	CAAGCTATAACGCAGCCTGTA
MERS-CoV	NP-F	CAAAACCTTCCCTAAGAAGGAAAAG
	NP-R	GCTCCTTTGGAGGTTCAGACAT
SARS-CoV	NP-F	ACCAGAATGGAGGACGCAAT
	NP-R	GCTGTGAACCAAGACGCAGTATTAT
H1N1	M-F	CTTCTAACCGAGGTCGAAACG
	M-R	GGC ATTTTGGACAAAKCGTCT A
H7N9	M-F	CTTCTAACCGAGGTCGAAACG
	M-R	GGC ATTTTGGACAAAKCGTCT A
Rhinovirus	5’UTR-F	AGCCYGCGTGGCKGCC
	5’UTR-R	AGCCYGCGTGGTGCCC
	Probe	HEX-TCCGGCCCCTGAATGYGGCTAA-lABkFQ
